# The Tipped Balance of ILC1/ILC2 in Peripheral Blood of Oral Lichen Planus Is Related to Inflammatory Cytokines

**DOI:** 10.3389/fcell.2021.725169

**Published:** 2022-01-31

**Authors:** Zi-Ming Wang, Jing Zhang, Fang Wang, Gang Zhou

**Affiliations:** ^1^ The State Key Laboratory Breeding Base of Basic Science of Stomatology (Hubei- MOST) and Key Laboratory of Oral Biomedicine Ministry of Education, School and Hospital of Stomatology, Wuhan University, Wuhan, China; ^2^ Department of Oral Medicine, School and Hospital of Stomatology, Wuhan University, Wuhan, China

**Keywords:** oral lichen planus, innate lymphoid cells, T cells, interleukin-12, interleukin-1β

## Abstract

Oral lichen planus (OLP) is an immune-inflammatory disease mediated by T cells. Innate lymphoid cells (ILCs) constitute a novel family of immune cells that initially originate from common innate lymphoid progenitors. Termed “T cells counterparts,” ILCs play a prominent role in inflammatory-immune diseases. However, the characterization of ILCs and their related induced factors were unclear in OLP. In the present study, the phenotypic characteristics of ILCs and their correlation with inflammatory cytokines were explored in the peripheral blood of OLP patients and healthy controls. We found that the proportion of total ILCs was expanded in OLP and was positively correlated with disease severity. The highly skewed distribution of ILC subpopulations was notable in OLP. Specifically, the frequency of ILC1s was significantly increased, while that of ILC2s was significantly reduced in total ILCs of OLP, resulting in the markedly elevated ILC1/ILC2 ratio in OLP. Correspondingly, ILCs in OLP displayed high expression of T-bet but low expression of GATA3. In addition, the IFN-γ expression level was elevated in ILC1s, whereas the IL-4 expression level was decreased in ILC2s. Moreover, ILC-associated activators IL-12, IL-18, and IL-1β were upregulated in OLP plasma, with IL-12 and IL-1β both positively correlated with the ILC1/ILC2 ratio. Further *in vitro* stimulation tests indicated that OLP plasma remarkedly increased the ILC1/ILC2 ratio, especially that IL-12 and IL-1β tipped the balance between ILC1s and ILC2s toward ILC1s in total ILCs. Overall, elevated levels of IL-12 and IL-1β might act as environmental cues in tipping the balance of ILC1/ILC2 in the peripheral blood of OLP, contributing to the immune dysregulation in OLP.

## Introduction

Oral lichen planus (OLP) is a common chronic inflammatory-immune disease affecting the oral mucosa with characteristic relapses and remissions (Farhi and Dupin, 2010; Alrashdan et al., 2016). The global prevalence was estimated at 2.2%, with a female predominance and a median age above 40 years (Carrozzo et al., 2019; Gonzalez-Moles et al., 2020). The WHO has identified OLP as a potentially malignant disorder with a malignant transformation rate from 0.07% to 5.8% (Van Der Waal, 2009; Aghbari et al., 2017). The etiology and pathogenesis of OLP remain unclear; however, it is generally acknowledged that T cell-mediated immune dysfunctions are responsible for the onset and progression of OLP (Nogueira et al., 2015). Extensive studies have elucidated that the immunopathogenesis of OLP may comprise T-cell proliferation, activation, and differentiation, as well as imbalanced cytokine network and activation of innate immune responses (Zhou et al., 2012; Adami et al., 2014; Lu et al., 2015). Especially, our previous studies revealed that OLP was featured by imbalanced T helper (Th)1/Th2 immune response and Th1/Th2 cytokine profile, with increased T-bet/GATA3 and IFN-γ/IL-4 ratios, which indicated the Th1 inclination for OLP dysimmunity (Lu et al., 2011; Zhou et al., 2012; Hu et al., 2013).

Termed “T lymphocytes counterparts,” innate lymphoid cells (ILCs) mirror Th1, Th2, and Th17/Th22 cells based upon their expression of transcription factors and cytokines but are very rare lineage negative cells that lack rearranged antigen-specific receptors (Vivier et al., 2018). ILCs constitute a novel family of immune cells that initially originate from common innate lymphoid progenitors and differentiate into three subpopulations with defined phenotypical and functional profiles: T-bet-dependent group 1 ILCs, including natural killer (NK) cells and IFN-γ-secreting ILC1; GATA-3-dependent group 2 ILCs (ILC2) that produce IL-4/IL-5/IL-13; and RORγt-dependent group 3 ILCs (ILC3) that secrete IL-17/IL-22 (Bando and Colonna, 2016; Klose and Artis, 2016; Vivier et al., 2018). As newly recognized immune cells, ILCs have emerging roles in regulating immunity, inflammation, and tissue homeostasis (Bando and Colonna, 2016; Klose and Artis, 2016; Vivier et al., 2018; Sonnenberg and Hepworth, 2019). Notably, ILCs express major histocompatibility complex (MHC) class II, simulating antigen-presenting cells to interact with T cells and thus modulating T-cell activation, proliferation, and polarization (Bando and Colonna, 2016; Sonnenberg and Hepworth, 2019). Additionally, ILCs are activated before T cells and rapidly respond to cytokines in their microenvironment, producing large amounts of inflammatory cytokines (Nagasawa et al., 2018; Sonnenberg and Hepworth, 2019). Thus, ILCs are crucial for orchestrating the innate and adaptive immune response.

Abnormal phenotypic characteristics of ILCs, which involve alterations in ILC phenotypes and function, have been observed in a wide range of immune-related inflammatory diseases in humans, including multiple sclerosis, rheumatic disease, psoriasis, and systemic lupus erythematosus (SLE) (Vivier et al., 2018; Xiong and Turner, 2018; Zhou et al., 2020). Guo et al. (2019) recently revealed that ILC1s were remarkably increased in the peripheral blood of SLE patients, whereas circulating ILC2s and ILC3s were decreased. Besides, several studies showed that ILC1s and ILC3s were expanded, ILC2s were reduced in lesional psoriatic skin, indicating that ILC1s and ILC3s might serve as pathogenic factors, and ILC2s might be protective factors in psoriasis (Xiong and Turner, 2018). ILCs have the capability to accommodate environmental cues by changing their phenotypic characteristics, and this plasticity is closely associated with the onset and progression of a broad spectrum of diseases (Bal et al., 2020; Zhou et al., 2020). Bal et al. (2016) reported in human airway inflammation in the lungs that ILC2s were directly activated by IL-1β and then were converted into IFN-γ-secreting ILC1s induced by IL-12, which was reversed by IL-4. These findings have suggested the immunological role of ILCs in human inflammatory-immune diseases. However, evidence for abnormal phenotypic characteristics of ILCs and their related induced factors in OLP remains unknown to date.

Herein, the present study firstly investigated phenotypical and functional profiles of ILC populations in the peripheral blood of OLP, analyzed their correlation with clinical types and disease severity, furtherly detected levels of signature ILC activators in OLP plasma, and performed *in vitro* plasma and cytokine stimulation tests, aiming to explore the phenotypic characteristics of ILCs and their correlation with inflammatory cytokines in OLP.

## Materials and Methods

### Study Subjects

A total of 45 OLP patients and 22 age- and gender-matched healthy controls were recruited from the Department of Oral Medicine, School and Hospital of Stomatology, Wuhan University. All the OLP cases enrolled in the study were in compliance with our previously described inclusion and exclusion criteria (Supplementary Table S1) (Peng et al., 2019). In addition, the OLP group was classified into two cohorts according to different clinical types: non-erosive OLP (NEOLP) and erosive OLP (EOLP). RAE (reticular, atrophic, and erosive lesion) scoring system was used to estimate the disease severity of OLP, by assessing 10 sites of OLP involvement in the oral cavity: the upper/lower labial mucosa, right buccal mucosa, left buccal mucosa, dorsal tongue, ventral tongue, floor of the mouth, hard palate mucosa, soft palate/tonsillar pillars, maxillary gingiva, and mandibular gingiva (Zhou et al., 2012). The control group consisted of nonsmoking, nonalcoholic healthy volunteers who were at least 18 years old and had no systemic disorders or any visible oral lesions. Based on the principles of the Declaration of Helsinki, written informed consent from each participant was obtained, and this study was approved by the Ethical Committee Board of the School and Hospital of Stomatology, Wuhan University. The clinical characterizations of the study participants are listed in Table 1.

**TABLE 1:** The clinical characterizations of the study participants.

Total number	OLP patient n = 45	Control n = 22
Gender		
Male	15	7
Female	30	15
Age (years)		
Range	21–69	24–63
Mean ± SD	46.84 ± 12.16	41.05 ± 13.93
Clinical form		
Non-erosive	23	
Erosive	22	
RAE scores		
Range	1–21	
Mean ± SD	7.54 ± 4.58	

Note. OLP, oral lichen planus; RAE, reticular, atrophic, and erosive lesion.

### Sample Preparation

The peripheral blood was collected in tubes precoated with EDTA-K_2_. Peripheral blood mononuclear cells (PBMCs) were isolated using Ficoll-Paque density gradient centrifugation. Harvested PBMC layer was washed twice and gently resuspended in phosphate-buffered saline (PBS). Then cell count and viability were tested by applying the Vi-CELL™ Cell Viability Analyzer (Beckman Coulter, Brea, CA, USA). Finally, 1 × 10^6^ cells were used in the following phenotypic analysis, and the remaining PBMCs were cryopreserved in liquid nitrogen for future tests. Plasma samples were centrifuged with a speed of 1,550 ×*g* for 15 min and stored at −80°C for later cytokine detection.

### Peripheral Blood Mononuclear Cells *In Vitro* Culture and Stimulation

In the plasma stimulation test, PBMCs from OLP patients and healthy controls were added in 12-well plates at 1 × 10^6^ cells per well and were cultured for 3 days in the presence of plasma from OLP patients or healthy controls. In cytokine stimulation test, PBMCs from OLP patients and healthy controls were added in 12-well plates at 1 × 10^6^ cells per well and were stimulated for 3 days with various combinations of 10 U/ml of IL-2, 50 ng/ml of IL-12, and 50 ng/ml of IL-1β (all from CHAMOTA, Shanghai, China) in Roswell Park Memorial Institute (RPMI) 1640 medium (Gibco Life Technologies, Grand Island, NY, USA) containing 10% fetal bovine serum (FBS; HyClone, Logan, UT, USA). To the end of stimulation, the collected PBMCs were processed with Fixable Viability Stain 700 (FVS700, BD Horizon™) dyeing for 20 min and subjected to flow cytometry.

### Multiparameter Flow Cytometric Analysis

Freshly isolated PBMCs were suspended in BD Pharmingen Stain Buffer (BSA; BD Biosciences, San Jose, CA, USA) and incubated with fluorochrome-conjugated antibodies (listed in Table 2) for 30 min in the dark at 4°C. Flow cytometry was performed on the CytoFLEX LX (Beckman Coulter, Fullerton, CA, USA), and FlowJo 10 (FlowJo LLC, Ashland, OR, USA) was applied to analyze flow cytometric data. ILCs were defined as CD45^+^ cells with high expression of CD127 and absence of lineage (Lin) markers (CD3, CD19, CD14, CD11b, CD11c, CD34, and CD56), which are for T, B, NK, and dendritic cells, as well as monocytes, macrophages, and stem cells. For further classification, CRTH2^+^ ILCs were considered to be ILC2s. CRTH2^−^CD117^−^ ILCs and CRTH2^−^CD117^+^ ILCs referred to ILC1s and ILC3s, respectively. ILC3s can be subdivided into natural cytotoxicity receptor (NCR)-negative ILC3s and NCR-positive ILC3s based on the expression of NKp44.

**TABLE 2:** Description of the antibodies used to define ILCs.

	Marker	Label	Source	Clone	Dilution
Lineage markers	CD3	FITC	BD	UCHT1	1/40
	CD19	FITC	BD	HIB19	1/40
	CD14	FITC	BD	M5E2	1/100
	CD11b	FITC	BD	M1/70	1/100
	CD11c	FITC	BD	B-ly6	1/100
	CD34	FITC	BD	581	1/40
	CD56	FITC	BD	B519	1/100
ILC surface markers	CD45	APC-Cy7	BD	2D1	1/200
	CD127 (IL7Ra)	PerCP-Cy5.5	BD	HIL-7R-M21	1/50
	CRTH2 (CD294)	PE-Cy7	BioLegend	BM16	1/50
	CD117 (cKit)	PE	BD	YB5.B8	1/50
	NKp44 (CD336)	APC	BD	p44-8	1/20
ILC intracellular markers	T-bet (TBX21)	PE-CF594	BD	O4-46	1/20
	GATA3	BV421	BD	L50-823	1/20
	RORγt	BV650	BD	Q21-559	1/20
	IFN-γ	BV605	BD	B27	1/20
	IL-4	BV786	BD	MP4-25D2	1/20
	IL-17A	BV510	BD	N49-653	1/20

Note. ILCs, innate lymphoid cells; FITC, fluorescein isothiocyanate.

Before intracellular staining, cells were activated for 6 h with 10 ng/ml of phorbol 12-myristate 13-acetate (PMA; Sigma, St. Louis, MO, USA), 500 nM of ionomycin (Sigma), and GolgiPlug protein transport inhibitor (Brefeldin A, BD Biosciences) in RPMI + 10% FBS medium. Then cells were fixed and permeabilized using a Cytofix/Cytoperm kit (BD Biosciences) according to the manufacturer’s instructions. Appropriate identification of ILC subpopulations was further evaluated by costaining with ILC fateful transcription factors T-bet, GATA3, and RORγt. Cytokine expression profiles were assessed by incubating with ILC signature cytokines IFN-γ, IL-4, and IL-17A.

### Enzyme-Linked Immunosorbent Assay

Cytokine (IL-12, IL-18, IL-25, IL-33, and IL-1β) levels in plasma from OLP patients and healthy controls were analyzed using ELISA kits (NeoBioscience Biological Technical Co. Ltd, Shenzhen, China) according to the manufacturer’s instructions. The absorbance at the 450-nm wavelength in each well was measured with Epoch Microplate Reader (BioTek, Winooski, VT, USA).

### Statistical Analysis

Statistical analysis was performed with GraphPad Prism 8.0 (GraphPad Software, San Diego, CA, USA), applying the Mann–Whitney U test, unpaired Student’s t-test, paired t-test, one-way ANOVA, and Spearman's correlation test. Experimental results were shown as mean ± SEM, and *p*-values less than 0.05 were considered statistically significant. For graphical rendering, R software v4.0.3 (www.r-project.org) was used in RStudio v 1.4.1103 environment (RStudio Inc., Boston, MA, USA), together with the ggplot2 package.

## Results

### Proportion of Total Innate Lymphoid Cells Was Expanded in the Peripheral Blood of Oral Lichen Planus

**FIGURE 1:**
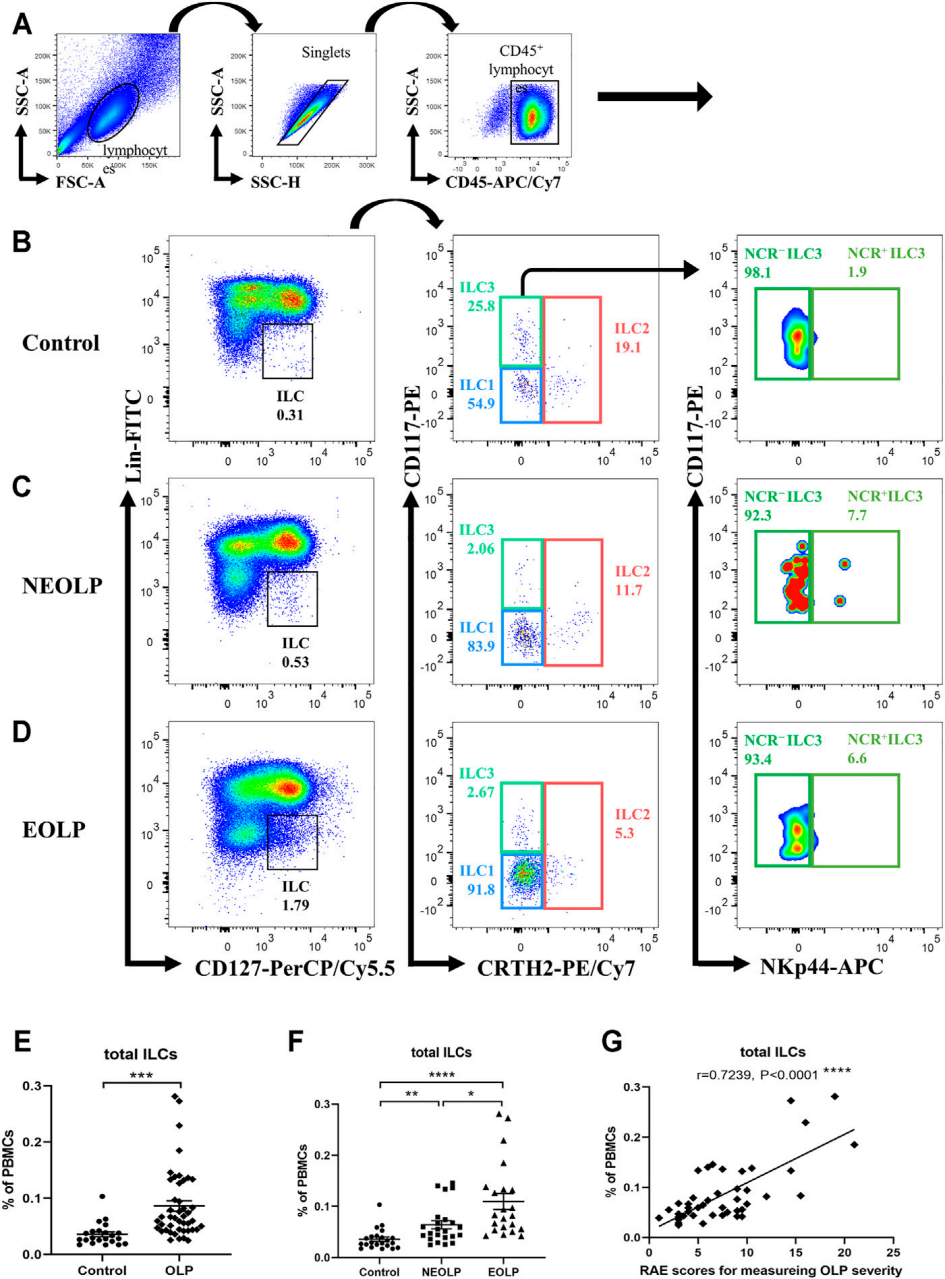
Identification of circulating ILCs. Isolated PBMCs from peripheral blood of OLP patients and healthy controls were stained for flow cytometric analyses. **(A–D)** Gating strategy. CD45^+^Lin^−^CD127^+^ cells were defined as ILCs. ILC2s were further gated on CRTH2^+^. CRTH2^−^CD117^−^ cells and CRTH2^−^CD117^+^ cells referred to ILC1s and ILC3s, respectively. NCR^−^ ILC3s and NCR^+^ ILC3s were gated based on the expression of NKp44. Representative plots of ILC populations in a healthy individual **(B)**, a NEOLP patient **(C)**, and an EOLP patient **(D)**. **(E,F)** The proportion of total ILCs in PBMCs. **(G)** Correlations of ILC proportion with corresponding disease severity in OLP. Data are depicted in correlation plots or as scatter dot plots with mean ± SEM. Two-tailed unpaired Mann–Whitney tests and Spearman’s rank correlation tests were performed, *p < 0.05, **p < 0.01, ***p < 0.001, and ****p < 0.0001. Lin, lineage; NCR, natural cytotoxicity receptor; OLP, oral lichen planus; NEOLP, non-erosive OLP; EOLP, erosive OLP; ILCs, innate lymphoid cells; PBMCs, peripheral blood mononuclear cells.

To better define the relative frequencies of circulating ILCs in OLP, we performed flow cytometric analysis to characterize total ILCs and ILC subpopulations in the peripheral blood of OLP patients. As shown in Figures 1A–D, a six-color staining panel was applied to gate total ILCs and ILC1/2/3. The frequency of total ILCs was significantly increased in OLP (*p* < 0.001, Figure 1E). And the same trends were found in different clinical types of OLP, as the NEOLP patients and EOLP patients both showed a notable expansion of total ILCs (NEOLP: *p* < 0.01, EOLP: *p* < 0.0001, Figure 1F). Clinical correlation analysis showed that the frequency of total ILCs in OLP was positively correlated with the corresponding OLP disease severity as measured by RAE scores (*p* < 0.0001, Figure 1G).

### Distribution of Innate Lymphoid Cell Subpopulations Was Altered in Oral Lichen Planus

**FIGURE 2:**
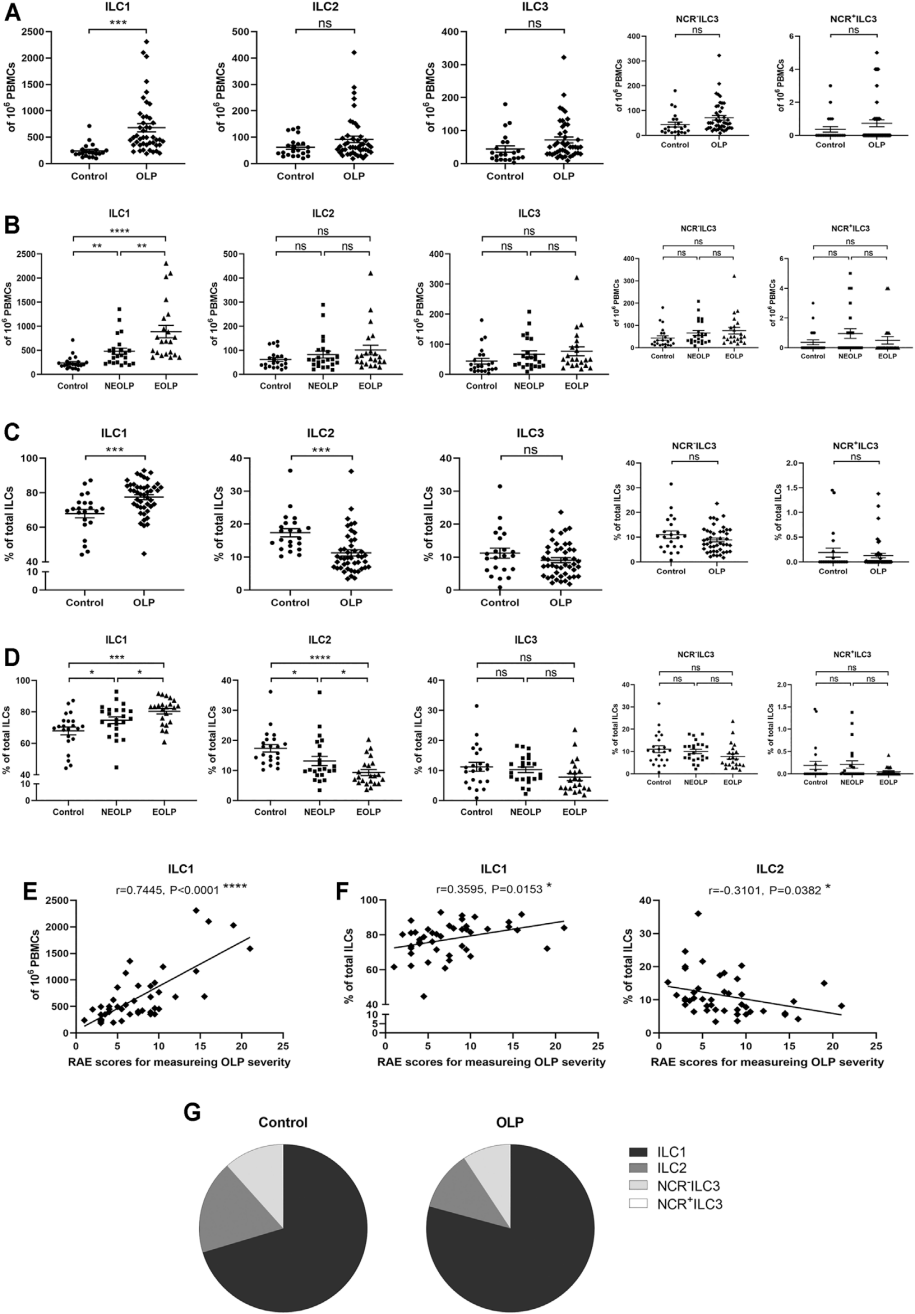
Distribution of ILC subpopulations. **(A,B)** The absolute numbers of ILC1s, ILC2s, and ILC3s (NCR^−^ ILC3s & NCR^+^ ILC3s) in 10^6^ PBMCs. **(C,D)** The proportions of ILC1s, ILC2s, and ILC3s (NCR^−^ ILC3s and NCR^+^ ILC3s) in total ILCs. Correlations of ILC1 counts in total PBMCs **(E)** as well as ILC1 and ILC2 proportions in total ILCs **(F)** with corresponding disease severity in OLP. **(G)** ILC distribution pie charts present the average percentage of each ILC subpopulation in total ILCs of the control group and OLP group. ILC, innate lymphoid cell; PBMCs, peripheral blood mononuclear cells; OLP, oral lichen planus.

The absolute number of ILC1s in OLP was dramatically increased (OLP: *p* < 0.001, Figure 2A; NEOLP: *p* < 0.01, EOLP: *p* < 0.0001, Figure 2B) and positively correlated with disease severity (*p* < 0.0001, Figure 2E), while other ILC subpopulations did not show significant difference (*p* > 0.05, Figures 2A,B). Of note, in total ILCs of OLP, the proportion of ILC1s (OLP: *p* < 0.001; NEOLP: *p* < 0.05, EOLP: *p* < 0.001) was dramatically increased, while the proportion of ILC2s was largely decreased (OLP: *p* < 0.001; NEOLP: *p* < 0.05, EOLP: *p* < 0.0001) (Figures 2C,D). There was also a tendency to lower proportions of ILC3s (NCR^−^ ILC3s & NCR^+^ ILC3s), but this difference did not reach significance (*p* > 0.05, Figures 2C,D). Furthermore, the rise of ILC1 proportion was positive, and the reduction of ILC2 proportion was negatively correlated with OLP severity (*p* < 0.05, Figure 2F). Figure 2G shows the distribution of different ILC subpopulations in the peripheral blood of OLP patients and healthy controls. ILC1s constituted the predominant ILC subpopulation in OLP, followed by ILC2s and NCR^−^ ILC3s, while NCR^+^ ILC3s were negligible in both groups.

**FIGURE 3:**
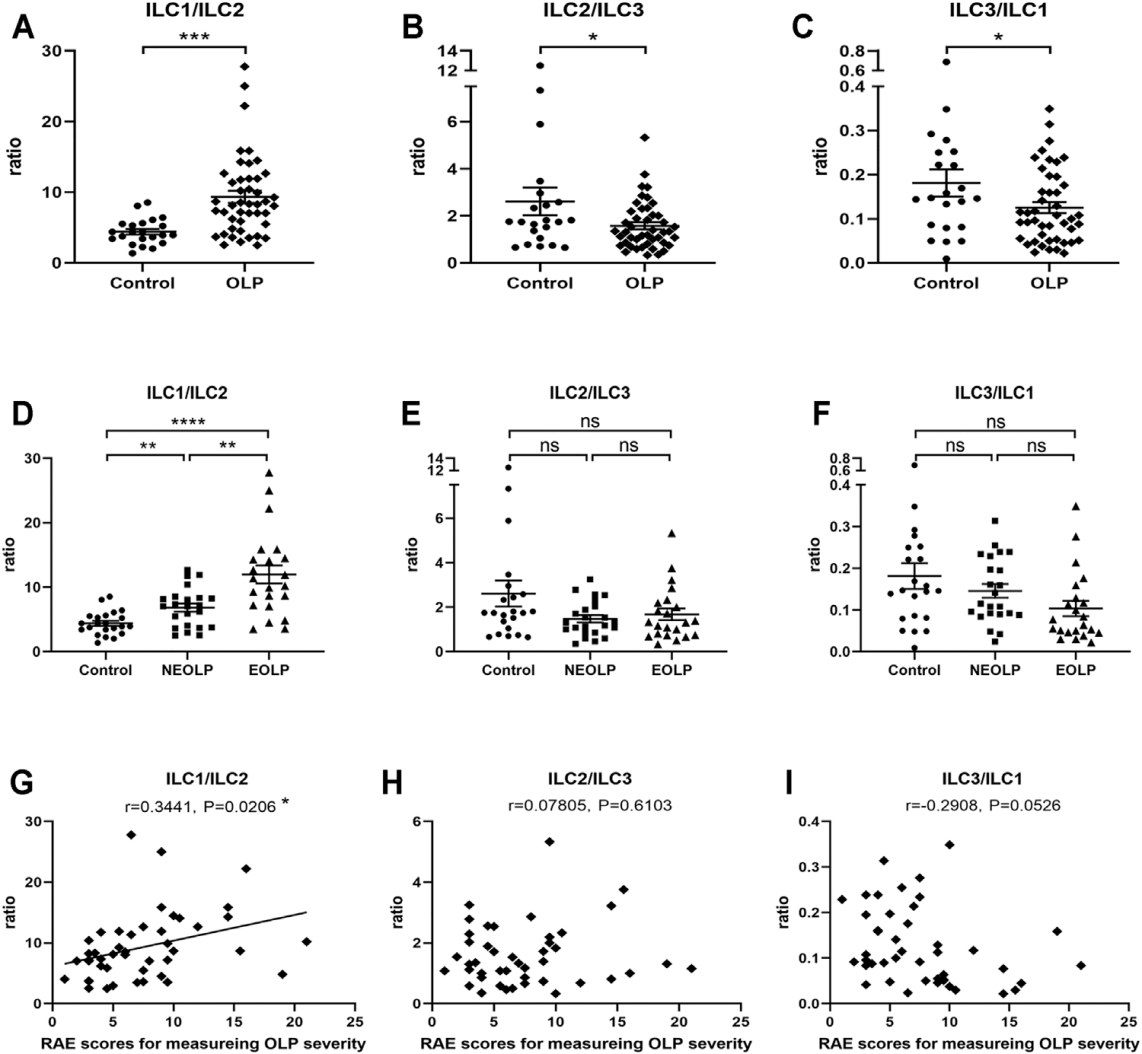
Altered ILC1/ILC2 balance in OLP. **(A–F)** The ratios of ILC1/ILC2, ILC2/ILC3, and ILC3/ILC1. **(G–I)** Correlation of ILC1/ILC3, ILC2/ILC3, and ILC3/ILC1 ratios with corresponding disease severity in OLP. OLP, oral lichen planus.

To initially explore the balance between each ILC subpopulation in OLP, we analyzed ILC1/ILC2, ILC2/ILC3, and ILC3/ILC1 ratios (Figure 3). The ratio of ILC1/ILC2 (*p* < 0.001) was markedly increased, while the ratios of ILC2/ILC3 (*p* < 0.05) and ILC3/ILC1 (*p* < 0.05) were significantly reduced in OLP (Figure 3A–C). Moreover, NEOLP patients (*p* < 0.01) and EOLP patients (*p* < 0.0001) also showed drastically higher ILC1/ILC2 ratios (Figure 3D), while the ratios of ILC2/ILC3 and ILC3/ILC1 in both types of OLP did not differ from those of the control group (*p* > 0.05, Figures 3E,F). As shown in Figure 3G, the ILC1/ILC2 ratio was positively correlated with disease severity in OLP (*p* < 0.05). The ratios of ILC2/ILC3 and ILC3/ILC1, however, did not correlate with OLP severity (*p* > 0.05, Figures 3H,I). These data demonstrated an altered balance between the proportions of ILC1s and ILC2s in OLP.

### Transcriptional and Cytokine Expression Profiles of ILC1s and ILC2s Were Changed in Oral Lichen Planus

**FIGURE 4:**
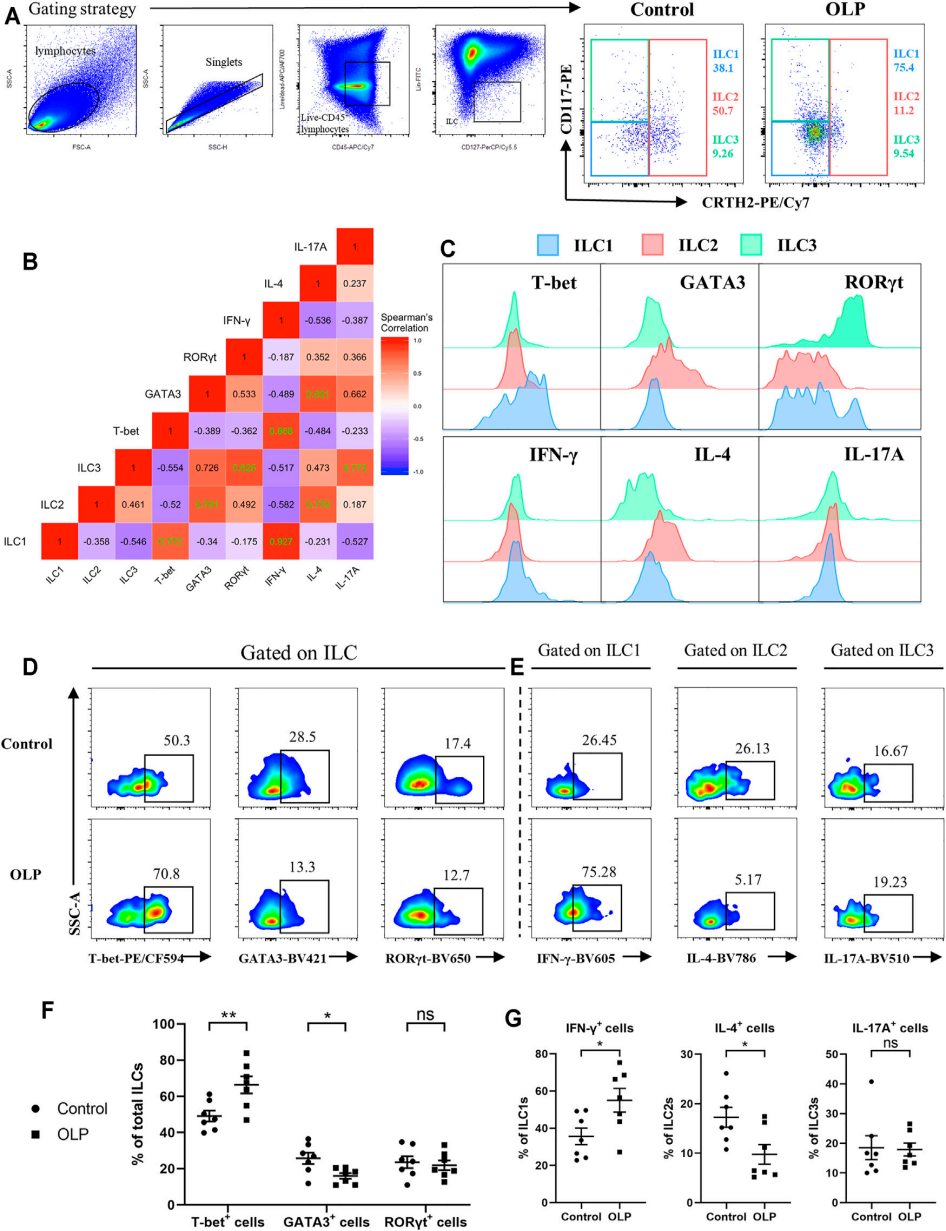
Transcriptional and cytokine expression profiles of ILCs. PBMCs collected from OLP patients and healthy controls were stimulated with PMA/ionomycin/Brefeldin A for 6 h and then subjected to intracellular staining and flow cytometric analyses. **(A)** Gating strategy to define ILC1/2/3. **(B)** Correlation heatmap depicting expression of transcription factors/cytokines in ILCs (Spearman’s correlation coefficient, healthy controls). Statistically significant correlations (two-tailed *p* < 0.05) are denoted in green. **(C)** Representative histograms of healthy individuals depicting transcriptional and cytokine expression profiles of ILC1/2/3. Representative FACS plots of expression of T-bet, GATA3, and RORγt in total ILCs **(D)** as well as expression of IFN-γ in ILC1s, expression of IL-4 in ILC2, and expression of IL-17A in ILC3 **(E)** among control and OLP groups. **(F)** Percentages of T-bet^+^, GATA3^+^, and RORγt^+^ cells in ILCs. **(G)** Percentages of IFN-γ^+^ cells in ILC1s, IL-4^+^ cells in ILC2s, and IL-17A^+^ cells in ILC3s. Data are depicted as a bar graph with mean ± SEM. Two-tailed unpaired Mann–Whitney tests were performed, *p < 0.05, **p < 0.01. ILCs, innate lymphoid cells; PBMCs, peripheral blood mononuclear cells; OLP, oral lichen planus; PMA, phorbol 12-myristate 13-acetate; FACS, fluorescence-activated cell sorting.

Human ILC1s, ILC2s, and ILC3s have been described as sharing parallel transcriptional and cytokine expression profiles with Th1, Th2, and Th17/22 cells, respectively (Vivier et al., 2018). Accordingly, intracellular staining of transcription factors and cytokines was performed to figure out functional alterations of ILC subpopulations in OLP (Figure 4A). The typical transcription factor and cytokine of each ILC population were further confirmed through analysis of correlation pattern, which determined co-expression of indicators in total ILCs of healthy controls (Figure 4B). In addition, expression levels of these transcription factors and cytokines in each ILC population were assessed, validating our gating strategy (Figure 4C).

As shown in Figures 4D,E, the percentage of T-bet^+^ cells was markedly increased (*p* < 0.01), while the percentage of GATA3^+^ cells was significantly decreased (*p* < 0.05) in total ILCs of OLP, implying an upregulation of T-bet accompanied by a downregulation of GATA3 in total ILCs of OLP (Figure 4F). Moreover, the percentage of IFN-γ^+^ cells in ILC1s was markedly increased, whereas the percentage of IL-4^+^ cells in ILC2s was significantly decreased (*p* < 0.05), suggesting a larger production of IFN-γ in ILC1s of OLP and a less production of IL-4 in ILC2s of OLP (Figure 4G). However, there was no difference in the expression of RORγt and IL-17A among ILCs of OLP (*p* > 0.05, Figures 4F,G).

### Levels of Innate Lymphoid Cell-Related Activation Factors Were Partly Increased in Oral Lichen Planus Plasma

**FIGURE 5:**
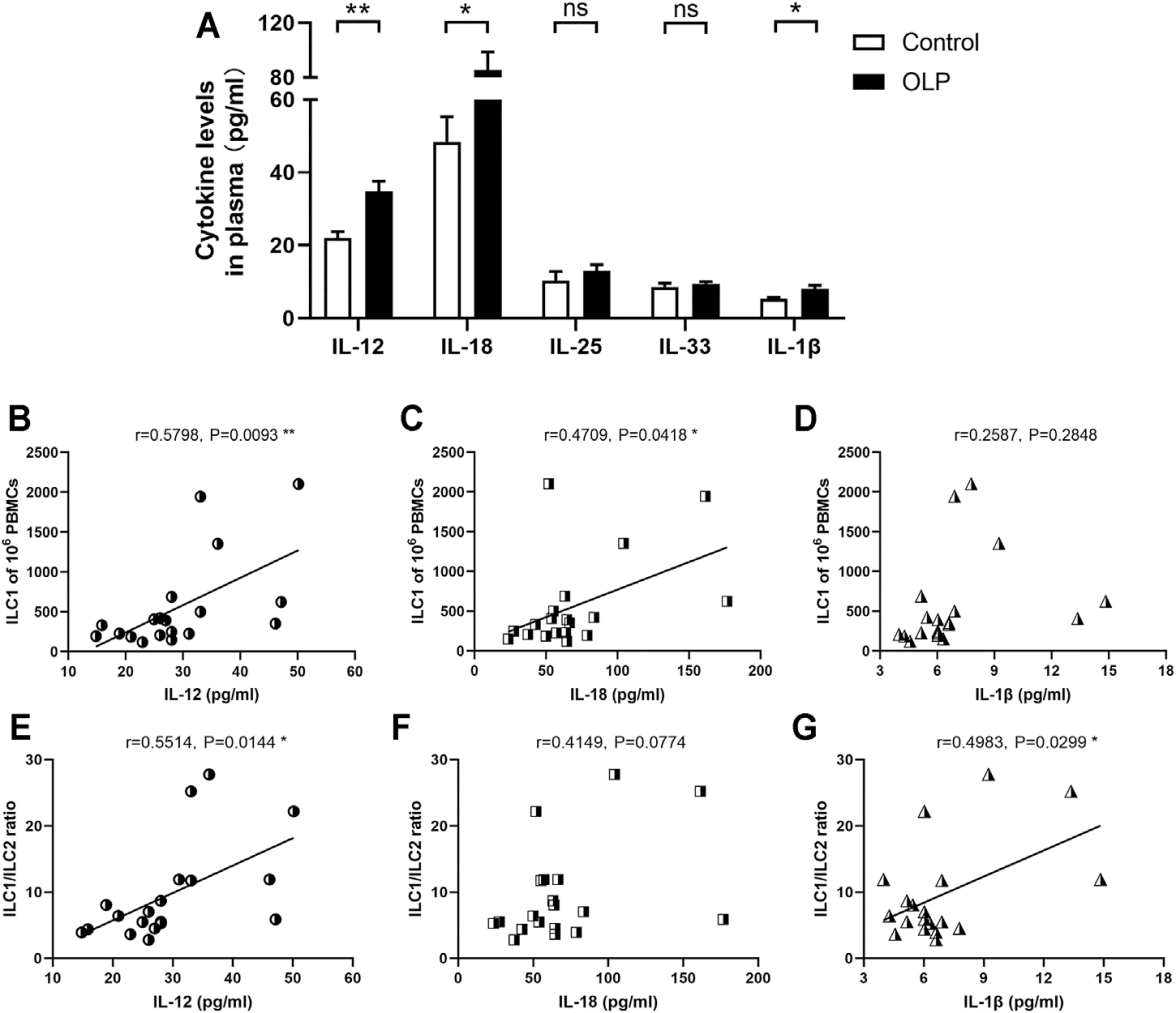
Plasma levels of ILC-related activation factors. Plasma extracted from OLP patients and healthy controls was measured by ELISA kits. **(A)** The expression levels of IL-12, IL-18, IL-25, IL-33, and IL-1β. Correlation of the expression levels of IL-12, IL-18, and IL-1β with corresponding circulating ILC1 counts **(B–D)** or ILC1/ILC2 ratio **(E–G)**. Data are depicted in correlation plots or as a bar graph with mean ± SEM. Two-tailed unpaired Mann–Whitney tests and Spearman’s rank correlation tests were performed, *p < 0.05, **p < 0.01. ILC, innate lymphoid cell; OLP, oral lichen planus.

IL-12, IL-18, IL-25, IL-33, and IL-1β have central roles in the regulation of ILCs (Nagasawa et al., 2018), and thus these cytokines were examined in plasma collected from OLP patients and healthy controls. As shown in Figure 5A, the expression levels of IL-12 (*p* < 0.01), IL-18 (*p* < 0.05), and IL-1β (*p* < 0.05) were significantly elevated in OLP plasma, while the plasma levels of IL-25 and IL-33 were similar between the OLP group and control group (*p* > 0.05).

Additionally, IL-12, IL-18, and IL-1β are critical activators that regulate the induction and plasticity of ILC subpopulations (Nagasawa et al., 2018). Therefore, the relations between plasma levels of these cytokines and the distribution of ILC subpopulations were evaluated. The levels of IL-12 (*p* < 0.01) and IL-18 (*p* < 0.05) were positively correlated with the absolute counts of ILC1s (Figure 5B–D). Furthermore, the levels of IL-12 and IL-1β were positively correlated with ILC1/ILC2 ratio (*p* < 0.05, Figure 5E–G).

### Oral Lichen Planus Plasma Stimulation Led to a Skewed Distribution of ILC1s and ILC2s

**FIGURE 6:**
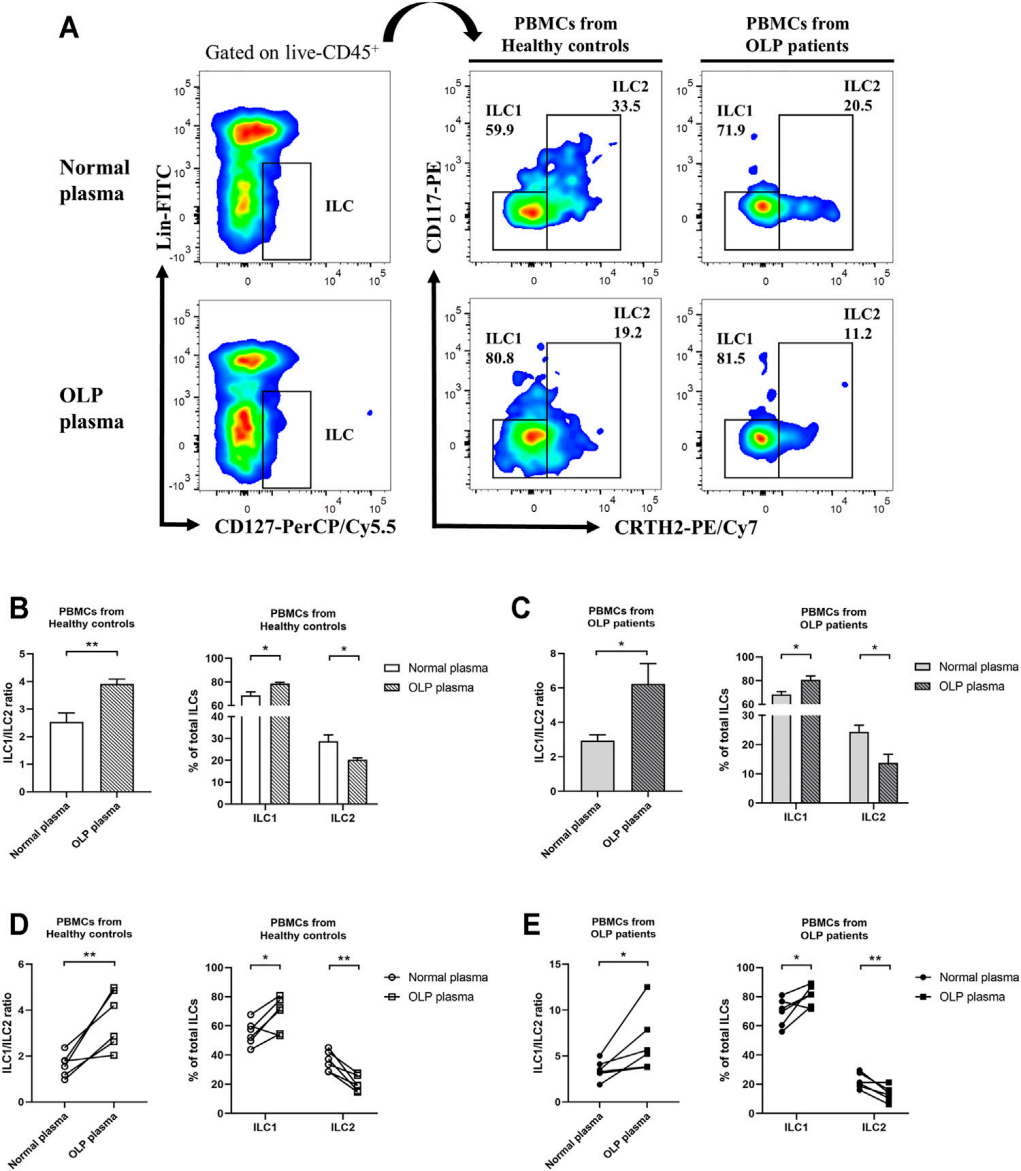
*In vitro* plasma stimulation test. PBMCs from healthy controls and OLP patients were stimulated with OLP plasma or normal plasma for 3 days. **(A)** Gating strategy identifying ILC1s and ILC2s. The ILC1 and ILC2 proportions in total ILCs as well as ILC1/ILC2 ratio were presented: PBMCs from a healthy individual **(B)** or an OLP patient **(C)** were stimulated with different OLP plasma or normal plasma separately for 3 days. PBMCs from different healthy individuals **(D)** or OLP patients **(E)** were stimulated with mixed OLP plasma or mixed normal plasma separately for 3 days. Data are depicted in before–after plots or as a bar graph with mean ± SEM. Two-tailed unpaired Mann–Whitney tests and paired t-tests were performed, *p < 0.05, **p < 0.01. PBMCs, peripheral blood mononuclear cells; OLP, oral lichen planus.

To investigate microenvironmental factors that were responsible for the altered distribution of ILC subpopulations, PBMCs from healthy controls and OLP patients were stimulated with OLP plasma or normal plasma (Figure 6A). In PBMCs from healthy controls, a drastically elevated ILC1/ILC2 ratio was observed after incubated with OLP plasma compared with normal plasma incubation (*p* < 0.01, Figures 6B,D). And the proportion of ILC1s was markedly increased, whereas the proportion of ILC2s was significantly reduced in total ILCs of healthy controls (*p* < 0.05, Figures 6B,D). In PBMCs from OLP patients, the same notable trend was found, as OLP plasma promoted a much more serious accumulation of ILC1s and depletion of ILC2s in total ILCs of OLP, leading to a largely increased ratio of ILC1/ILC2 (*p* < 0.05, Figures 6C,E).

### IL-12/IL-1β Stimulation Tipped the Balance of ILC1/ILC2 Toward ILC1s

**FIGURE 7:**
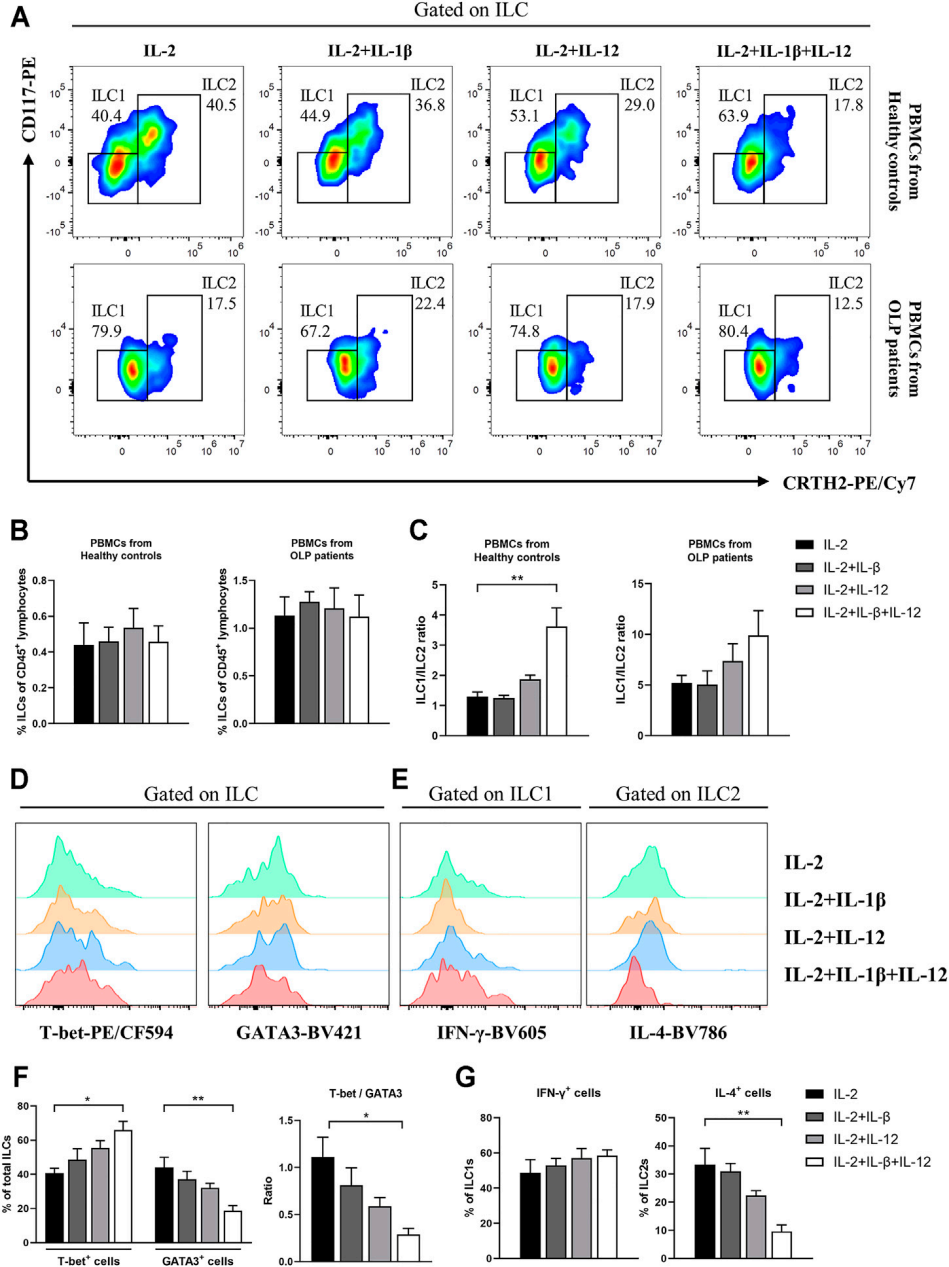
*In vitro* IL-12/IL-1β stimulation test. PBMCs collected from healthy controls and OLP patients were stimulated with IL-2 alone or with IL-2 and various combinations of IL-1β and IL-12 for 3 days. **(A)** Representative FACS plots defining ILC1s and ILC2s. As shown in healthy controls and OLP patients: **(B)** percentages of total ILCs in CD45^+^ lymphocytes; **(C)** ratio of ILC1/ILC2. As shown in healthy controls: representative histograms of intracellular staining for T-bet and GATA3 in ILCs **(D)** as well as IFN-γ in ILC1s and IL-4 in ILC2s **(E)**; **(F)** percentages of T-bet^+^ and GATA3^+^ cells as well as ratio of T-bet/GATA3 in total ILCs; **(G)** percentages of IFN-γ^+^ cells in ILC1s and IL-4^+^ cells in ILC2s. Data are depicted as a bar graph with mean ± SEM. One-way ANOVA was performed, *p < 0.05, **p < 0.01. PBMCs, peripheral blood mononuclear cells; OLP, oral lichen planus; FACS, fluorescence-activated cell sorting.

Based on the above results of cytokine level detection in plasma and plasma stimulation test, we sought to determine whether IL-12 and IL-1β were able to induce these abnormal phenotypic characteristics of ILC1s and ILC2s in OLP (Figure 7A). In PBMCs from healthy controls or OLP patients, the frequencies of circulating ILCs were similar between subgroups that received different stimuli (*p* > 0.05, Figure 7B). Notably, in PBMCs from healthy controls, the combination of IL-12 and IL-1β induced a specifically elevated ratio of ILC1/ILC2 (*p* < 0.01), while IL-12 or IL-1β alone did not induce this change (*p* > 0.05) (Figure 7C). In PBMCs from OLP patients, however, there was no distinct variation of ILC1/ILC2 ratio observed in different subgroups (*p* > 0.05, Figure 7C).

Figures 7D,E display the intracellular staining analyses of ILCs from healthy controls. IL-12/IL-1β drove a higher expression of T-bet (*p* < 0.05) and lower expression of GATA3 (*p* < 0.01) in total ILCs, which caused an elevated T-bet/GATA3 ratio (*p* < 0.05, Figure 7F). In addition, the relative IL-4 expression in ILC2s was dramatically reduced (*p* < 0.01), while no significant effect of IL-12/IL-1β stimulus on IFN-γ expression in ILC1s was observed (*p* > 0.05) (Figure 7G). These findings probably highlighted the non-negligible roles of microenvironmental IL-12 and IL-1β that modulate the balance between ILC1s and ILC2s in circulating ILCs.

## Discussion

OLP is an immune-inflammatory disease mediated by T cells. ILCs constitute a novel family of immune cells that initially originate from common innate lymphoid progenitors. Being the counterparts of T cells, ILCs are emerging as vital regulators of immunity, inflammation, and tissue homeostasis (Klose and Artis, 2016; Vivier et al., 2018; Sonnenberg and Hepworth, 2019). However, the phenotypical and functional profiles of ILCs in OLP remain poorly investigated. Herein, we first demonstrated that the phenotypic characteristics of ILCs were dramatically altered with an elevated expression of ILC1 and reduced expression of ILC2 in the peripheral blood of OLP patients. Moreover, the plasma levels of ILC-associated activators IL-12, IL-18, and IL-1β were largely increased in OLP. Especially, the *in vitro* stimulation of IL-12 and IL-1β tipped the balance between ILC1s and ILC2s toward ILC1s in circulating ILCs.

Accumulating evidence has described that the altered proportions of total ILCs were found in various human diseases, and different ILC subpopulations could play a critical role in immunity and inflammatory diseases (Xiong and Turner, 2018). In this study, the overall frequency of ILCs in OLP was substantially increased. And the distribution of ILC subpopulations was highly skewed, as the proportion of ILC1s was expanded while the proportion of ILC2s was reduced in total ILCs of OLP. Therefore, it is possible that ILCs might be engaged in the pathogenesis of OLP. Soare et al. (2018) reported the highly significant correlation of the ILC2/ILC3 ratio with disease activity indexes in psoriatic arthritis, suggesting the potential of ILC as a biomarker for immunological disease activity. Here, correlation analyses showed the strongest positive association between ILC1 counts and corresponding RAE scores in OLP (*r* = 0.7445, *p* < 0.0001), indicating that circulating ILC1s might serve as a promising molecular predictor of OLP disease severity.

Interestingly, the present study found that the expression of T-bet in total ILCs of OLP was upregulated, and the production of IFN-γ in greatly enriched ILC1s of OLP was enhanced. Bernink et al. (2013) revealed that the frequency of circulating ILC1s was elevated in intestinal injury and ILC1-derived IFN-γ exaggerated inflammation. Furthermore, Okabayashi et al. (2019) reported that treatment with ILC1-targeted receptor agonist attenuated renal injury by downregulating T-bet mRNAs expression and reducing IFN-γ production. Through IFN-γ secretion, ILC1s can aid Th1 cell priming and modulate Th1 immune response directly or indirectly (Sonnenberg and Hepworth, 2019). In OLP, the Th1 immune response plays a leading role, and a high level of IFN-γ has been recognized (Zhou et al., 2012; Hu et al., 2015). Our recent work found that IFN-γ level regulated by elevated miR-29b in OLP consequently modulated Th1 immune response *via* a feedback loop (Zhang et al., 2020). Overall, we suggested that activated ILC1s, which promptly secrete IFN-γ and are essential for the initiation and regulation of Th1 immune response, might contribute to the pathogenetic process of OLP.

In contrast, the current research showed that the expression of GATA3 in total ILCs of OLP was downregulated, and the production of IL-4 in largely depleted ILC2s of OLP was weakened. A requisite role for ILC2s in regulating adaptive immunity has been well documented, as activated ILC2s can enhance CD4^+^ T-cell function and potently drive Th2 differentiation in an IL-4-dependent manner, which subsequently triggers type 2 immunity (Pelly et al., 2016). Our previous study demonstrated that the level of IL-4, which is the representative cytokine of Th2 cells, declined in serum and lesions of OLP patients (Zhou et al., 2012; Lu et al., 2015). It is also noteworthy that Cao et al. recently revealed that treatment with ILC2-activator IL-33 in mouse models induced large amounts of IL-4 production in ILC2s and a renoprotective effect of ILC2s in ischemia–reperfusion injury (Cao et al., 2018). Based on our findings, we speculated that the activity of ILC2s was suppressed in OLP, and ILC2s might serve as protective factors in the immunity and inflammation of OLP.

It is well established that OLP was featured by imbalanced Th1/Th2 immune response and Th1/Th2 cytokine profile, with increased T-bet/GATA3 and IFN-γ/IL-4 ratios, which indicated the Th1 inclination for OLP dysimmunity (Lu et al., 2011; Zhou et al., 2012; Hu et al., 2013). The present data of tipped balance of ILC1/ILC2 in OLP might consequently contribute to this trend. Although ILCs are “counterparts” of Th cells, ILCs respond rapidly to environmental signals in an antigen-independent fashion through their early generation during the formation of the immune system (Bando and Colonna, 2016; Nagasawa et al., 2018; Sonnenberg and Hepworth, 2019). The aberrant expression patterns of inflammation-related cytokines in OLP have been widely delineated. In this study, we detected levels of ILC-related activation factors in OLP plasma and found that the expression levels of IL-12, IL-18, and IL-1β, which were dramatically elevated in OLP plasma, were consistent with that reported in OLP studies (Zhang et al., 2012; Huang et al., 2016; Seebauer et al., 2020). Relation analysis showed that the plasma levels of IL-12 and IL-18 were positively correlated with the ILC1 counts, which supported that ILC1s are preferentially activated by IL-12 and IL-18 (Nagasawa et al., 2018). Besides, the plasma levels of IL-12 and IL-1β were positively correlated with ILC1/ILC2 ratio, implying that environmental signals potentially distorted the ILC1/ILC2 balance in the immunity of OLP.

Like Th cells, the phenotypic characteristics of ILC subpopulations are unstable, and these cells accordingly adapt to changes in cytokine milieu by altering their function and related phenotypes (Dupage and Bluestone, 2016; Elemam et al., 2017). This plasticity has been well charted in ILC research and factors involved in ILC plasticity have also been identified (Bal et al., 2020). A research on SLE reported that plasma from SLE patients potentiated the IFN-γ production in ILC1s (Guo et al., 2019). In the present study, the *in vitro* stimulation of OLP plasma not only increased the ratio of ILC1/ILC2 in healthy controls but also caused a higher proportion of ILC1s and a lower proportion of ILC2s in total ILCs of OLP, which might lead to the occurrence and protracted course of OLP. Ohne et al. (2016) reported that IL-1β potently promoted ILC2 activation and plasticity in human airway inflammation, and IL-12 switched ILC2s into IFN-γ-secreting ILC1s. The present data exhibited that ILC1/ILC2 ratio was largely elevated in PBMCs of healthy control after being stimulated with IL-12 and IL-1β *in vitro*; nevertheless, the frequency of total ILCs did not show a difference. Moreover, the production of IL-4 in ILC2s was decreased, while the production of IFN-γ in ILC1s was not affected. Our findings indicated that IL-12 and IL-1β mainly acted on ILC2s as well as changed the phenotype and function of ILC2s, which might drive the conversion of ILC2s into ILC1s. No statistical difference was found in the PBMCs of OLP, implying that more complicated factors induced the imbalance of ILC1/ILC2 in the progression of OLP. Bernink et al. (2015) revealed that transdifferentiation of pathogenic ILC1s to protective ILC3s driven by cytokine stimulation in inflammatory bowel disease ameliorated the disease symptoms. Taken together, it is reasonable to hypothesize that the tipped balance of ILCI/ILC2 in OLP was associated with the plasticity of ILC2s regulated by environmental IL-12 and IL-1β, which might help to develop innovative therapeutic targets and treatment strategies for OLP.

Although increasing data indicated an overexpression pattern and selectively regulatory role of IL-23/IL-17 axis in the pathogenesis of OLP (Lu et al., 2014), the expression of Th17-like ILC3s in OLP did not show an obvious difference in this research. This may be due to the rare frequency of ILC3s in the peripheral blood and their plasticity to transdifferentiate into ILC1s or derive from ILC2s in inflamed tissue (Bal et al., 2020). Moreover, this study focused on the ILC populations in the peripheral blood of OLP. Lots of research has demonstrated that peripheral immunity might play an important part in OLP (Lu et al., 2011; Zhang et al., 2017). From a different perspective, characterized as tissue-resident cells, ILCs might not only act locally but also recirculate to remote target tissue *via* the blood (Klose and Artis, 2016). Thus, we would like to begin with research on the ILC populations in the peripheral blood of OLP and may focus on distinct ILC populations in local OLP lesions in future research.

**FIGURE 8:**
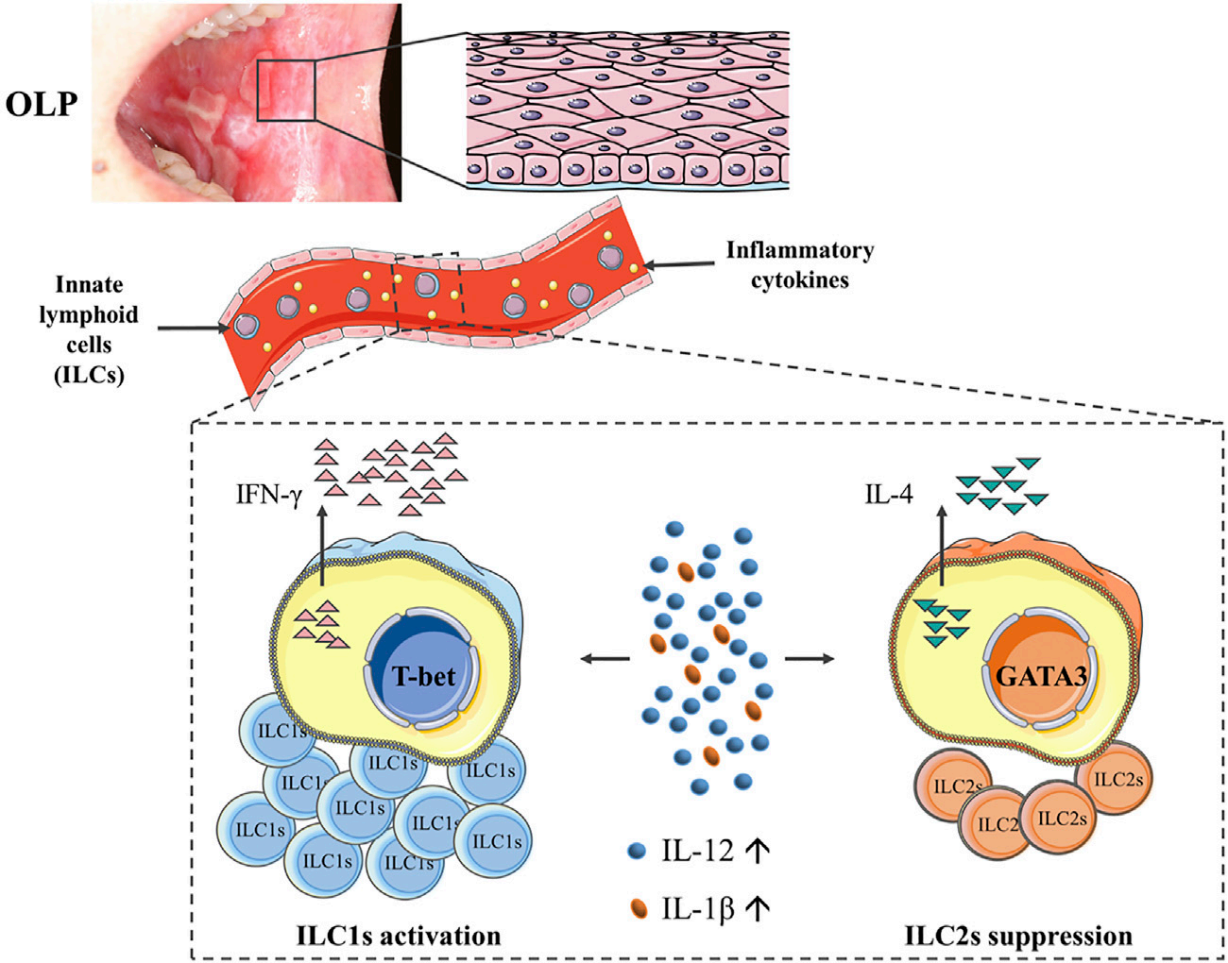
Diagram illustrating that the phenotypes and function of circulating ILCs in OLP were altered, showing an imbalanced ILC1/ILC2 state with ILC1 activation and ILC2 suppression. Of note, elevated levels of IL-12 and IL-1β might act as environmental cues in tipping the balance of ILC1/ILC2 in peripheral blood of OLP, thus contributing to the immune dysregulation in OLP. ILCs, innate lymphoid cells; OLP, oral lichen planus.

In conclusion, our study was the first to demonstrate the alterations of phenotypes and function of circulating ILCs in OLP, showing a skewed balance of ILC1/ILC2 with ILC1 activation and ILC2 suppression. Of note, elevated levels of IL-12 and IL-1β might act as environmental cues in tipping the balance of ILC1/ILC2 in the peripheral blood of OLP, thus contributing to the immune dysregulation in OLP (Figure 8). Further studies are warranted to validate the molecular mechanisms of ILCs in OLP.

## Data Availability

The original contributions presented in the study are included in the article/Supplementary Material. Further inquiries can be directed to the corresponding author.
