# Supplementary material for: The Tipped Balance of ILC1/ILC2 in Peripheral Blood of Oral Lichen Planus Is Related to Inflammatory Cytokines
Source: Front Cell Dev Biol. 2022 Jan 31;9:725169. doi: 10.3389/fcell.2021.725169 (PMC8842723; doi:10.3389/fcell.2021.725169)
Supplement: Supplementary file 1 [file Table1.DOCX]

Supplementary Material

# Supplementary Table 1

The inclusion and exclusion criteria for oral lichen planus (OLP) patients

| ***The inclusion criteria:*** |
| --- |
| 1. At least 18 years of age. |
| 2. Clinical presence of symmetric white striations. Erosive, atrophy, blisters, papules, and plaques were only accepted as a subtype.  3. Histopathological presence of a band‐like predominantly lymphocytes infiltration in the lamina propria with basal cell degeneration. |
| ***The exclusion criteria:*** |
| 1. A history of smoking or alcohol abuse. |
| 2. Pregnancy, lactation. |
| 3. Subject with infectious, allergic, cardiovascular, hematological, endocrine, metabolic, and immune-related diseases. |
| 4. Exposure to systemic or topical anti-inflammatory, immunomodulatory drugs at least within 3 months. |
| 5. Patient with concomitant other oral lesions. |
| 6. Participant with oral lichenoid reactions, including lichenoid contact reactions, lichenoid drug eruptions, and lichenoid reactions of graft-versus-host disease. |
| 7. Presence of epithelial dysplasia in histopathological examination. |
